# Efficacy and safety of dorzagliatin for type 2 diabetes mellitus: A meta-analysis and trial sequential analysis

**DOI:** 10.3389/fcvm.2022.1041044

**Published:** 2022-11-23

**Authors:** Yunfeng Yu, Xingyu Yang, Keke Tong, Shuang Yin, Gang Hu, Fei Zhang, Pengfei Jiang, Manli Zhou, Weixiong Jian

**Affiliations:** ^1^College of Chinese Medicine, Hunan University of Chinese Medicine, Changsha, China; ^2^The First Affiliated Hospital of Hunan University of Chinese Medicine, Changsha, China; ^3^The Hospital of Hunan University of Traditional Chinese Medicine, Changde, China

**Keywords:** dorzagliatin, type 2 diabetes mellitus, meta-analysis, trial sequential analysis, harbord

## Abstract

**Objective:**

To evaluate the efficacy and safety of dorzagliatin in the treatment of type 2 diabetes mellitus (T2DM) by using meta-analysis and trial sequential analysis (TSA).

**Method:**

Search for clinical trials of dorzagliatin for T2DM in eight databases, with a time limit of build to July 2022. The included studies that met the requirements were carried out for meta-analysis and TSA.

**Results:**

In terms of efficacy endpoints, meta-analysis showed that dorzagliatin decreased glycated hemoglobin A1c(HbA1c) [mean difference (MD) −0.65%, 95% confidence interval (CI) −0.76 ~ −0.54, *P* < 0.00001], fasting plasma glucose (FPG) (MD −9.22 mg/dL, 95% CI −9.99 ~ −8.44, *P* < 0.00001), 2 h postprandial glucose (2h-PPG) (MD −48.70 mg/dL, 95% CI −55.45 ~ −41.96, *P* < 0.00001), homeostasis model assessment 2 of insulin resistance (HOMA2-IR) (MD −0.07, 95% CI −0.14 ~ −0.01, *P* = 0.03) and increased homeostasis model assessment 2 of ß-cells function (HOMA2-β) (MD 2.69, 95% CI 1.06 ~ 4.31, *P* = 0.001) compared with placebo. And TSA revealed that the benefits observed for the current information set were conclusive, except for HOMA2-IR. In comparison with placebo, dorzagliatin increased triglyceride(TG) (MD 0.43 mmol/L, 95% CI 0.30 ~ 0.56, *P* < 0.00001), total cholesterol (TC) (MD 0.13 mmol/L, 95% CI 0.05 ~ 0.21, *P* = 0.001), body weight (MD 0.38 kg, 95% CI 0.12–0.63, *P* = 0.004) and body mass index (BMI) (MD 0.14 kg/m^2^, 95% CI 0.05–0.24, *P* = 0.003), while low density lipoprotein cholesterol (LDL-C), high-density lipoprotein cholesterol (HDL-C), systolic blood pressure (SBP) and diastolic blood pressure (DBP) were comparable. And TSA demonstrated that TG, TC, body weight, and BMI were conclusive. In terms of safety endpoints, dorzagliatin increased total adverse events (AEs) [risk ratio (RR) 1.56, 95% CI 1.06 ~ 2.30, *P* = 0.03], while serious AEs, hyperlipidemia, and hypoglycaemia were all comparable. And TSA indicated that the results need to be confirmed by additional studies. Harbord regression showed no publication bias.

**Conclusion:**

Dorzagliatin was effective in lowering glycemia, reducing insulin resistance and improving islet ß-cells function without affecting blood pressure, LDL-C, and HDL-C. Although dorzagliatin caused a mild increase in TG and TC, it did not increase the incidence of hyperlipidemia, and the small increases in body weight and BMI were not clinically significant enough. In terms of safety, the total AEs caused by dorzagliatin may be a cumulative effect of single AEs, with no drug-related adverse event being reported at a higher incidence than placebo alone. Dorzagliatin's serious AEs, hyperlipidemia, and hypoglycemia are comparable to that of placebo, and dorzagliatin has a good safety profile.

**Systematic review registration:**

https://www.crd.york.ac.uk/prospero/display_record.php?RecordID=371802 identifier: CRD42022371802.

## Introduction

Type 2 diabetes mellitus (T2DM) is a progressive chronic metabolic disease characterized by pancreatic ß-cells dysfunction and insulin resistance (1). Epidemiology has shown that about 537 million adults worldwide had diabetes in 2021, 90–95% of whom had T2DM, and the prevalence of T2DM is still increasing (2). T2DM can induce retinal, renal and cardiovascular complications (3, 4), and it is part of the leading causes of death and disability worldwide (5), which seriously affects the physical and mental health of diabetic patients (6). Currently, the treatment of T2DM relies on the combination of multiple hypoglycemic agents or insulin (7), but there are still a small number of patients with poor glycemic control and an increasingly prominent risk of adverse events (AEs). The onset of T2DM is associated with an increase of hepatic glucose output, and metformin is the only drug commonly used in clinical settings that can target a reduction in hepatic glucose output (8). However, metformin has a limited ability to lower glycemia and often needs to be combined with other hypoglycemic agents (9). Glucokinase (GK) acts as a glucose sensor with a role in regulating glucose-stimulated insulin secretion (10), and promotes hepatic glucose uptake as well as glycogen synthesis and storage (8), thereby maintaining glycemic homeostasis in patients. GK has become a popular target for hypoglycemic drug research, however, the development and research of previous glucokinase agonist (GKA) has not been smooth. GKA such as piragliatin and ARRY-403 were terminated due to the poor hypoglycemic effect and the risk of inducing hypoglycemia and increasing liver burden (11), while novel GKA are still in the development stage.

Dorzagliatin is a novel hypoglycemic agent for the treatment of T2DM with dual agonistic effects on GK in the pancreas and liver (12). It can promote the secretion of insulin and the synthesis of hepatic glycogen, improve the utilization of glucose by peripheral tissues (7), and also improve the function of β-cells in patients with T2DM (13). Dorzagliatin has been reported to significantly and consistently reduce glycated hemoglobin A1c (HbA1c) in patients with T2DM and is well-tolerated (14). Currently, no meta-analysis related to dorzagliatin is available, and the evidence-based evidence of dorzagliatin for T2DM remains to be elucidated. Therefore, this study was conducted to search for published randomized controlled trials of dorzagliatin for T2DM and to carry out meta-analysis and trial sequential analysis (TSA) in order to provide an evidence-based basis for the clinical use of dorzagliatin.

## Methods

This study strictly followed the systematic review and meta-analysis methodology of the Preferred Reporting Items for Systematic reviews and Meta-Analyses (PRISMA) (15).

### Search strategy

The China National Knowledge Infrastructure (CNKI, https://www.cnki.net/), Chinese Biology Medicine (CBM, http://www.sinomed.ac.cn/index.jsp), VIP (http://qikan.cqvip.com/), WanFang (https://www.wanfangdata.com.cn/), Embase (https://www.embase.com/), PubMed (https://pubmed.ncbi.nlm.nih.gov/), the Cochrane Library (https://www.cochranelibrary.com/), and Web of Science (https://www.webofscience.com/) databases were searched for clinical studies of dorzagliatin in the treatment of T2DM, all with a time limit from database creation to July 2022. The English subject headings cover dorzagliatin, type 2 diabetes mellitus. The Chinese subject headings cover dorzagliatin, erxing tangniaobing (the Chinese name of type 2 diabetes mellitus). Based on the subject terms, we expanded the Chinese free terms with the help of CKNI and CBM databases, and the English free terms with the help of MeSH database, and then combined the subject terms and free terms for searching.

### Inclusion and exclusion criteria

We followed the inclusion criteria (shown below). (1) Type of data: Randomized controlled trial. (2) Participants: Meet the basic diagnosis of T2DM (16). (3) Intervention: Patients in the experimental group were treated with dorzagliatin and the control group with placebo or other hypoglycemic agents. (4) Indicators: HbA1c was used as the primary efficacy endpoint. Fasting plasma glucose (FPG), 2 h post-prandial glucose (2h-PPG), homeostasis model assessment 2 of ß-cells function (HOMA2-β), homeostasis model assessment 2 of insulin resistance (HOMA2-IR), systolic blood pressure (SBP), diastolic blood pressure (DBP), triglyceride (TG), total cholesterol (TC), low density lipoprotein cholesterol (LDL-C), high-density lipoprotein cholesterol (HDL-C), body weight, body mass index (BMI) were used as secondary efficacy endpoints. Total AEs, serious AEs, hyperlipidemia, hypoglycaemia were used as safety endpoints. All of the safety endpoints were drug-related.

We guided by the exclusion criteria (as follows). (1) Studies such as reviews, animal studies, and case reports. (2) Studies with repeated publications. (3) Studies with incomplete data. (4) Studies that did not use intervention blinding of participants.

### Literature screening, data statistics, and risk of bias

In the first step, the basic literature retrieved from each database was imported into Endnote X9, and the irrelevant literature was eliminated after reading the title, abstract and full text in turn according to the inclusion and exclusion criteria, finally identifying the included literature. In the second step, the included literature was categorized, and basic characteristics such as author, year, sample size, mean age, sex ratio, intervention, and course of treatment were extracted and entered into the data statistical table. In the third step, the risk of bias was assessed using the Cochrane risk of bias assessment tool based on the requested entries. All work was conducted independently by two investigators, and any disagreement was resolved by a third investigator.

### Statistical analysis

We used Revman5.3 to conduct meta-analysis, and risk ratio (RR) and 95% confidence interval (95% CI) were used as effect sizes for dichotomous variables. Continuous variables used mean difference (MD) and 95% CI as effect sizes. Heterogeneity was analyzed by *I*^2^*-*test and *Q*-test. If *I*^2^ < 50% and *P* > 0.1, the heterogeneity was small and fixed-effect model (FEM) analysis was used. Otherwise, random effects model (REM) analysis was used. The TSA0.9.5.10 Beta software was employed for the TSA, and the original results were conclusive if the cumulative *Z*-value crossed the required information size (RIS) or TSA bound. Publication bias was assessed by using Stata15.0 software. If the scatter on both sides of the funnel plot was essentially symmetrical and the harbord regression showed *P* > 0.1, there was no publication bias. We chose to evaluate the quality of the evidence by using GRADEpro3.6 software, and the evaluation method was based on the GRADE evidence evaluation guidelines.

## Results

### Literature search result

**Figure 1:**
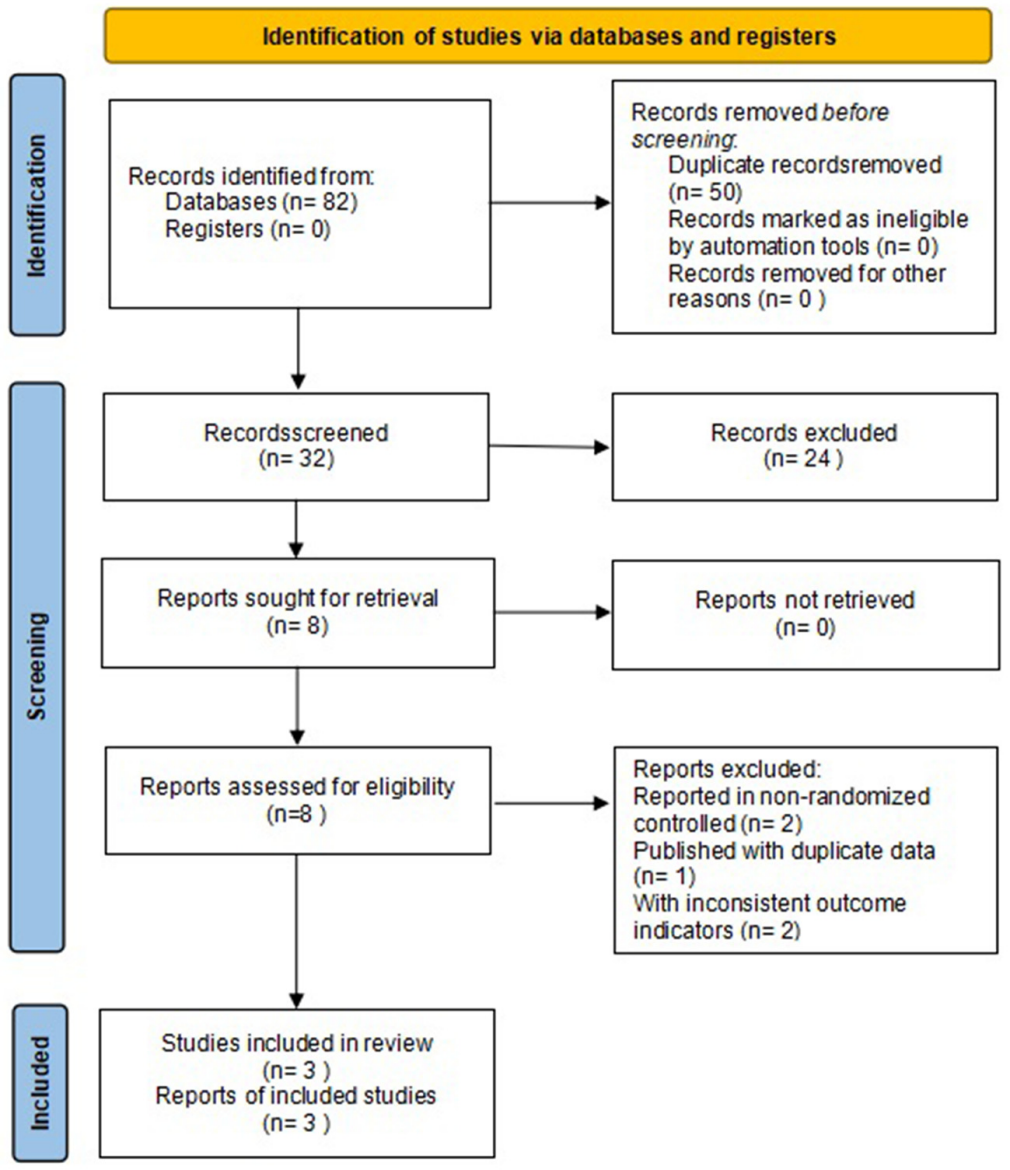
Flow chart of literature search.

A total of 82 studies were retrieved, 50 duplicates were screened out, 24 were excluded after reading the titles and abstracts, five were excluded after reviewing the full text, and 3 were finally included (17–19). The flow chart of literature search is shown in Figure 1.

### Basic information

A total of 3 clinical studies were included (17–19) with a total sample size of 1,332 cases, including 584 cases in the dorzagliatin group and 748 cases in the placebo group. The study centers were all located in China, and the basic characteristics of the included studies are shown in Table 1.

**Table 1:** Table of basic characteristics of included studies.

**References**	**Patient number**	**Treatment duration (weeks)**	**Intervention**	**Number randomized**	**Disease duration (years)**	**Age (years)**	**Male *N*/(%)**
Yang et al. (19)	767	24	Dorzagliatin 75 mg bid Metformin 1,500 mg qd	382	6.14	54.6 ± 10.0	245 (64)
			Placebo bid Metformin 1,500 mg qd	385	5.78	54.4 ± 9.2	230 (60)
Zhu et al. (18)	463	24	Dorzagliatin 75 mg bid	153	1	53.2 ± 9.6	200 (65)
			Placebo bid	310	0.89	53.5 ± 10.0	101 (66)
Zhu et al. (17)	102	24	Dorzagliatin 75 mg bid	49	3.41	55.4 ± 7.7	31 (63)
			Placebo bid	53	3.37	54.7 ± 8.5	31 (58)

### Risk of bias assessment

**Figure 2:**
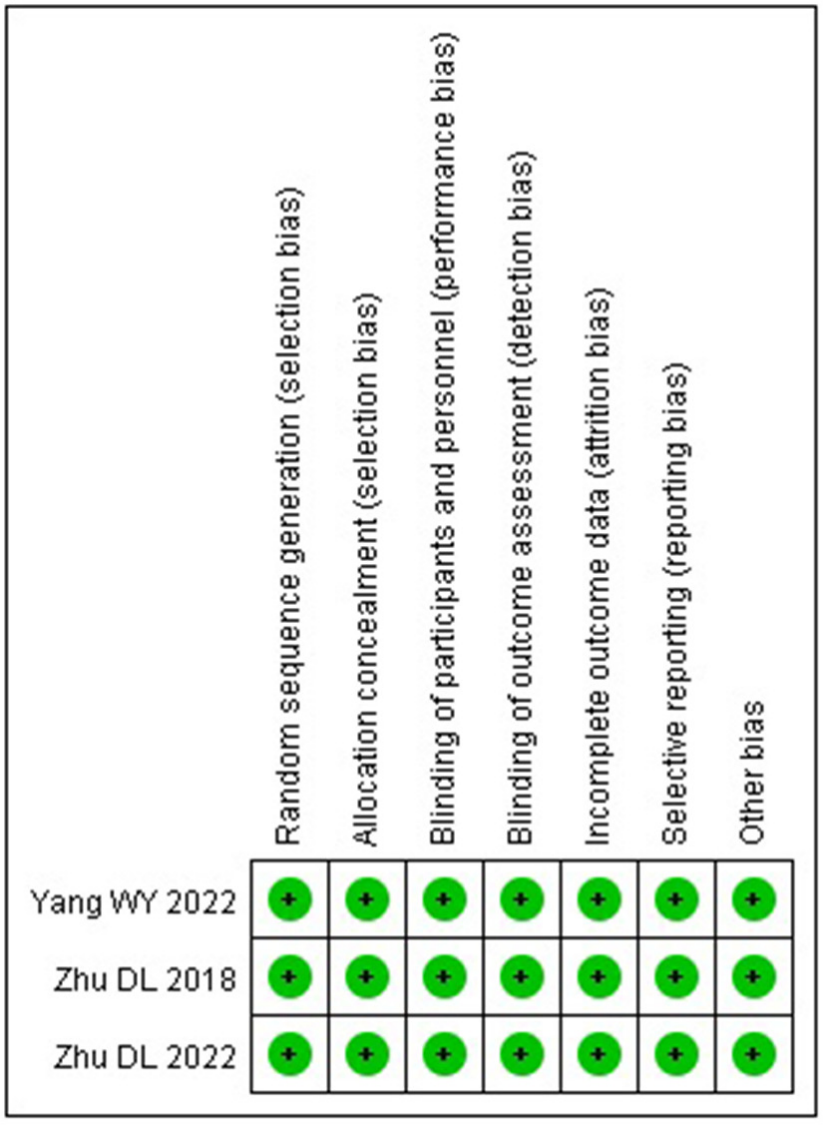
Graph of risk bias of included studies.

The three included studies (17–19) were all high-quality studies with a low risk of bias in all aspects. The risk of bias of the included studies is shown in Figure 2.

### Efficacy endpoint

#### Glycemic-Related endpoints

**Figure 3:**
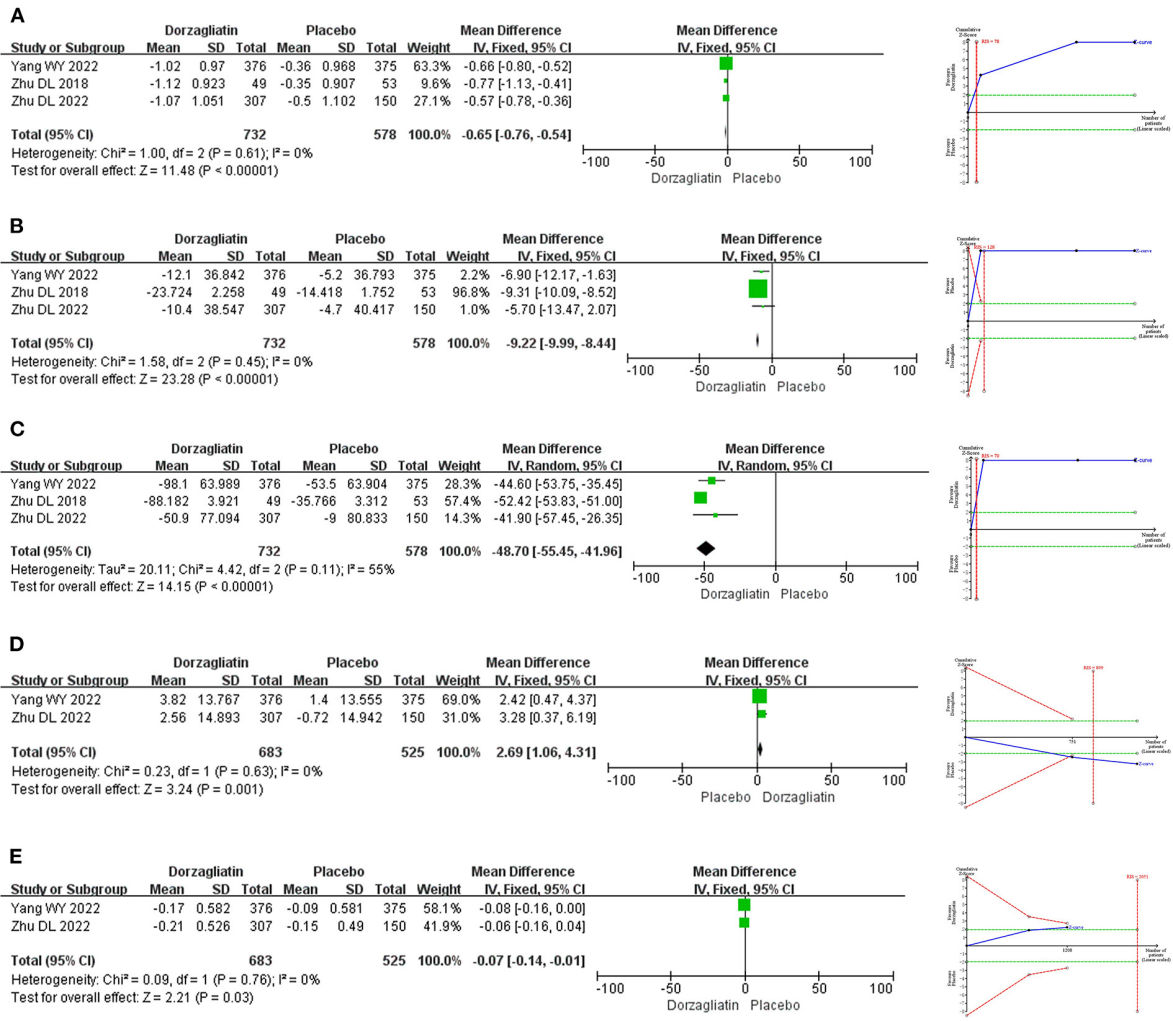
Meta-analysis and TSA results of glycemic-related indicators in dorzagliatin vs. placebo in the treatment of T2DM. **(A)** Meta-analysis and TSA results of HbAlc in dorzagliatin vs. placebo in the treatment of T2DM. **(B)** Meta-analysis and TSA results of FPG in dorzagliatin vs. placebo in the treatment of T2DM. **(C)** Meta-analysis and TSA results of 2h-PPG in dorzagliatin vs. placebo in the treatment of T2DM. **(D)** Meta-analysis and TSA results of HOMA2-β in dorzagliatin vs. placebo in the treatment of T2DM. **(E)** Meta-analysis and TSA results of HOMA2-IR in dorzagliatin vs. placebo in the treatment of T2DM.

Meta-analysis revealed that dorzagliatin significantly reduced HbA1c by 0.65% (MD −0.65, 95% CI −0.76 ~ −0.54, *P* < 0.00001), FPG by 9.22 mg/dL (MD −9.22, 95% CI −9.99 ~ −8.44, *P* < 0.00001), 2h-PPG by 48.70 mg/dL (MD −48.70, 95% CI −55.45 ~ −41.96, *P* < 0.00001), and HOMA2-IR by 0.07 (MD −0.07, 95% CI −0.14 ~ −0.01, *P* = 0.03) and significantly increased HOMA2-β by 2.69 (MD 2.69, 95% CI 1.06 ~ 4.31, *P* = 0.001) when compared with placebo. TSA indicated that the benefits observed for the current information set were conclusive, with the exception of HOMA2-IR. The GRADE evaluation showed high quality of evidence for HbA1c, FPG, and HOMA2-β, and moderate quality of evidence for 2h-PPG and HOMA2-IR. As shown in Figure 3.

#### Blood pressure-related endpoints

**Figure 4:**
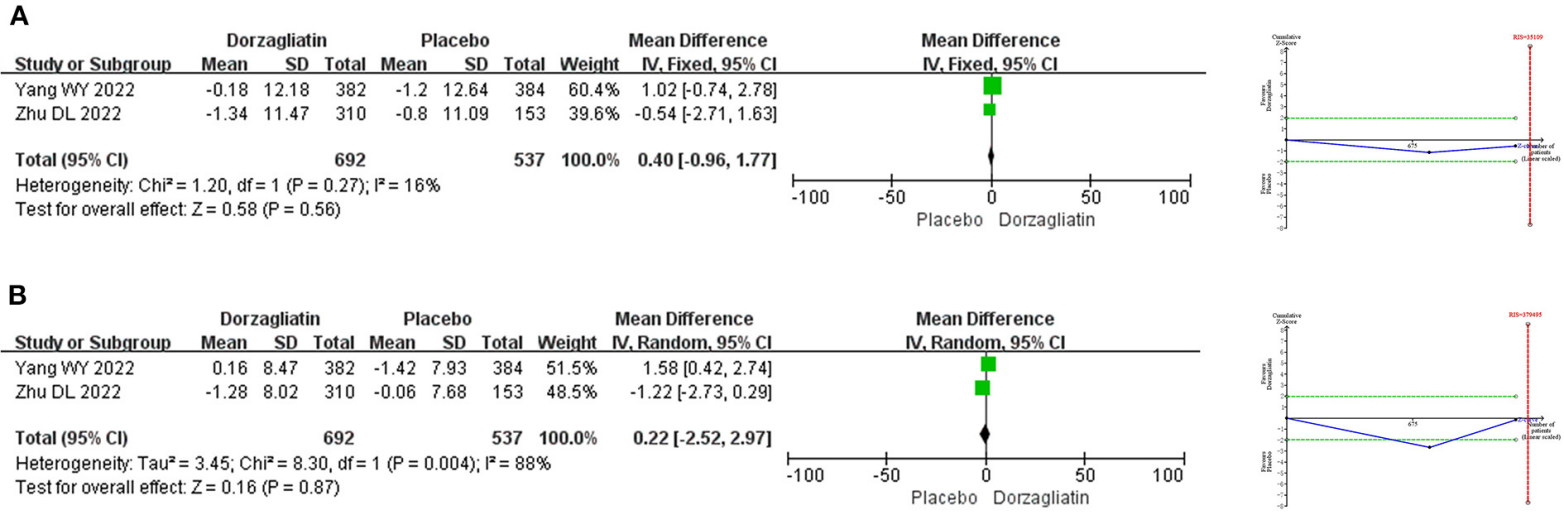
Meta-analysis and TSA results of blood pressure-related indicators in dorzagliatin vs. placebo in the treatment of T2DM. **(A)** Meta-analysis and TSA results of SBP in dorzagliatin vs. placebo in the treatment of T2DM. **(B)** Meta-analysis and TSA results of DBP in dorzagliatin vs. placebo in the treatment of T2DM.

Meta-analysis demonstrated that the SBP (MD 0.40, 95% CI −0.96 to 1.77, *P* = 0.56) and DBP (MD 0.22, 95% CI −2.52 ~ 2.97, *P* = 0.87) were comparable for dorzagliatin compared with placebo. TSA suggested a RIS of 35,109 for SBP and 379,495 for DBP, implying that the current results need to be validated by more studies. The GRADE evaluation showed a moderate quality of evidence for SBP and a low quality of evidence for DBP. As shown in Figure 4.

#### Blood lipid-related endpoints

**Figure 5:**
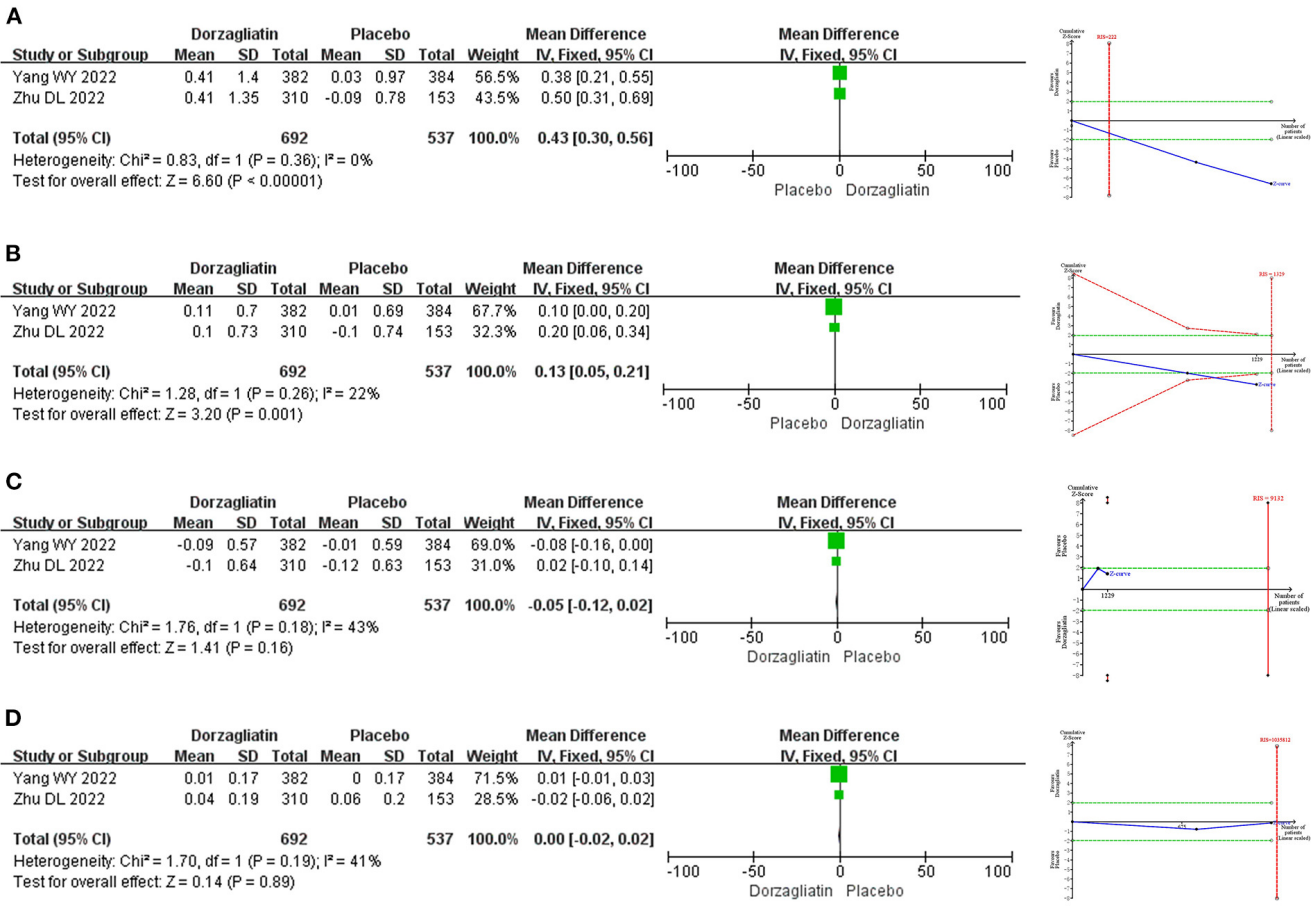
Meta-analysis and TSA results of blood lipid-related indicators in dorzagliatin vs. placebo in the treatment of T2DM. **(A)** Meta-analysis and TSA results of TG in dorzagliatin vs. placebo in the treatment of T2DM. **(B)** Meta-analysis and TSA results of TC in dorzagliatin vs. placebo in the treatment of T2DM. **(C)** Meta-analysis and TSA results of LDL-C in dorzagliatin vs. placebo in the treatment of T2DM. **(D)** Meta-analysis and TSA results of HDL-C in dorzagliatin vs. placebo in the treatment of T2DM.

Meta-analysis demonstrated that dorzagliatin increased TG by 0.43 mmol/L (MD 0.43, 95% CI 0.30 ~ 0.56, *P* < 0.00001) and TC by 0.13 mmol/L (MD 0.13, 95% CI 0.05 ~ 0.21, *P* = 0.001) compared with placebo, while LDL-C (MD −0.05, 95% CI −0.12 ~ 0.02, *P* = 0.16) and HDL-C (MD 0.00, 95% CI −0.02 ~ 0.02, *P* = 0.89) were not statistically different. TSA indicated that differences in TG and TC were conclusive, while differences in LDL-C (RIS = 1,035,812) and HDL-C (RIS = 9,132) need to be confirmed by additional studies. The GRADE evaluation showed high quality of evidence for TG and TC, and moderate quality of evidence for LDL-C and HDL-C. As shown in Figure 5.

#### Body weight-related endpoints

**Figure 6:**
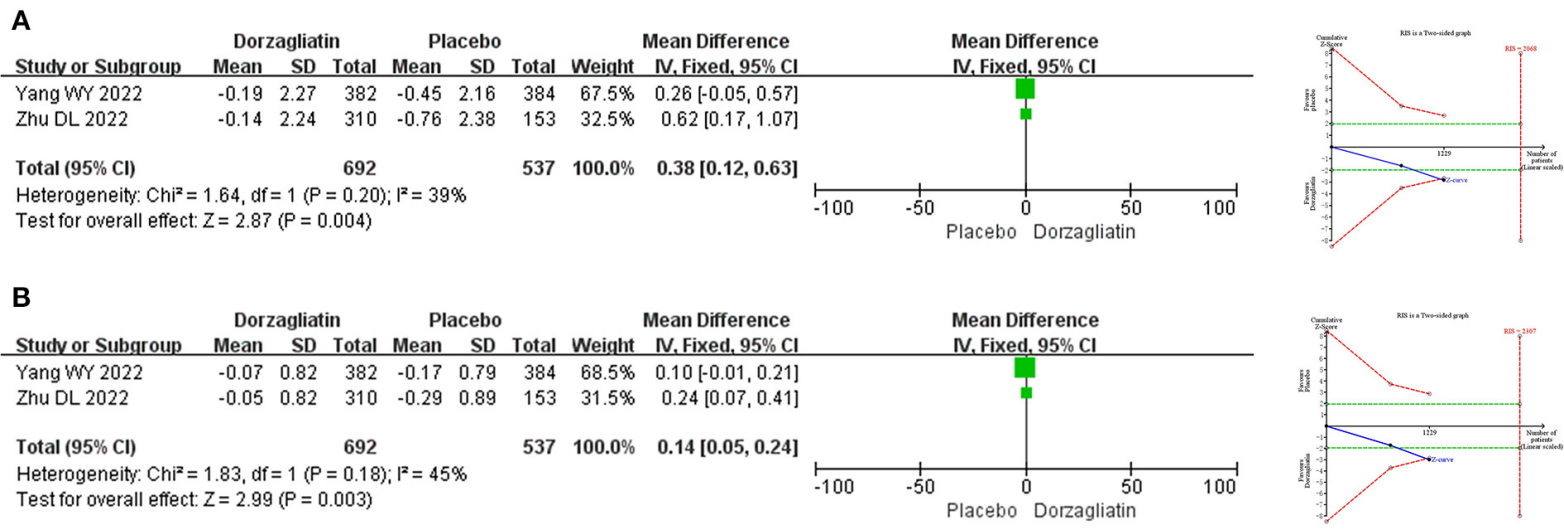
Meta-analysis and TSA results of body weight-related indicators in dorzagliatin vs. placebo in the treatment of T2DM. **(A)** Meta-analysis and TSA results of body weight in dorzagliatin vs. placebo in the treatment of T2DM. **(B)** Meta-analysis and TSA results of BMI in dorzagliatin vs. placebo in the treatment of T2DM.

Meta-analysis indicated that dorzagliatin increased body weight by 0.38 kg (MD 0.38, 95% CI 0.12–0.63, *P* = 0.004) and BMI by 0.14 kg/m^2^ (MD 0.14, 95% CI 0.05–0.24, *P* = 0.003) compared with placebo. TSA revealed that the results observed for the current information set were conclusive. The GRADE evaluation showed high quality of evidence for both body weight and BMI. As shown in Figure 6.

### Safety endpoint

Meta-analysis demonstrated that dorzagliatin increased total AEs (RR 1.56, 95% CI 1.06 ~ 2.30, *P* = 0.03) by 56% compared with placebo, while serious AEs (RR 0.17, 95% CI 0.01 ~ 4.03, *P* = 0.27), hyperlipidemia (RR 1.80, 95% CI 0.27 ~ 12.18, *P* = 0.55), and hypoglycaemia (RR 3.64, 95% CI 0.65 ~ 20.37, *P* = 0.14) were all comparable. TSA suggested that none of these results observed in the current informative set were conclusive and needed to be confirmed by more relevant studies. The GRADE evaluation showed a moderate quality of evidence for all of these indicators. As shown in Table 2.

**Table 2:** Meta-Analysis and TSA results of dorzagliatin for AEs.

**Outcome**	**Dorzagliatin arm** **(events/total)**	**Placebo arm** **(events/total)**	***I^2^***	**RR (95% CI)**	**TSA**	**Quality of evidence**
Total AEs	59/743	37/590	0	1.56 (1.06, 2.30)	No	Moderate
Serious AEs	0/692	1/537	0	0.17 (0.01, 4.03)	No	Moderate
Hyperlipidemia	3/692	1/537	0	1.80 (0.27, 12.18)	No	Moderate
Hypoglycaemia	5/743	0/590	0	3.64 (0.65, 20.37)	No	Moderate

### Publication bias

**Figure 7:**
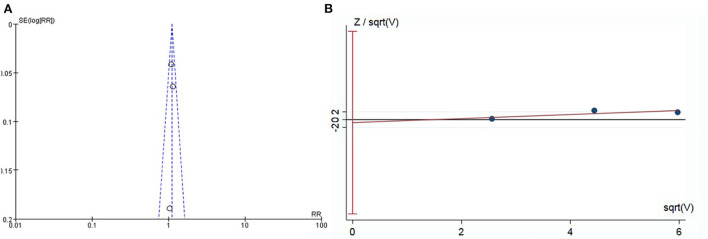
Publication bias assessment graph. **(A)** Funnel plot. **(B)** Harbord regression.

Funnel plots indicated basic symmetry of the scatter on both sides, and harbord regression of total AEs showed no significant publication bias (*P* = 0.75; Figure 7).

## Discussion

GKA is a new class of hypoglycemic agents that lower glycemia by activating pancreatic and hepatic GK, regulating glucose-stimulated insulin secretion (20) and promoting hepatic glycogen synthesis (8). Unfortunately, a series of GKA development and clinical trials have not yet been successful. Piragliatin was the first GKA to enter clinical studies (21) and rapidly lowered FPG and postprandial glucose in patients with T2DM in a dose-dependent manner (22), but was later found to increase the risk of potential lactic acidosis, fatty liver, and hypoglycemia (23–25). AZD-1656, another GKA, was effective in reducing HbA1c in patients with T2DM, but its hypoglycemic effect diminished over time, with no significant difference in HbA1c reduction from placebo at the fourth month (26). In addition, AZD-1656 has been reported to independently increase triglycerides by 4–22% (27), as well as induce hypoglycemia, and no further studies related to AZD-1656 have been published since 2014 to date (28). ARRY-403, a dual pancreatic and hepatic GK agonist, is effective in reducing FPG and postprandial glucose in patients with T2DM who are poorly controlled metformin (29). However, its hypoglycemic incidence was as high as 9.0% (29), thus limiting the clinical use of ARRY-403. The risk of hypoglycemia and elevated blood lipids are the main conflicts that exist in GKA and are the main reasons for GKA to discontinue clinical trials. GKA-related drug development and clinical trials are still ongoing, and researchers are continuing to explore a new type of hypoglycemic drug that combines efficacy and safety.

Dorzagliatin, the first GKA to enter phase III clinical trials (30), has dual agonistic effects on pancreatic and hepatic GK. It not only promotes insulin secretion from pancreatic β-cells, but also promotes glycogen synthesis and storage in the liver, thereby promoting peripheral glucose uptake and utilization and exerting hypoglycemic effects. Studies have shown that dorzagliatin has a good hypoglycemic effect and can effectively reduce HbA1c, FPG and 2h-PPG, which has potential value of application (31). However, given that GKA tends to increase the incidence of hypoglycemia and has a risk of elevated lipids in previous studies, its safety remains to be further evaluated. This meta-analysis and TSA included a total of three clinical studies and 1,332 sample sizes and is the first publication to date to examine dorzagliatin for T2DM, with the aim of assessing the specific benefits and potential risks of dorzagliatin for T2DM. TSA, harbord regression and quality of evidence evaluation gave a more comprehensive and credible result for this study.

HbA1c is formed by glycosylation of hemoglobin and mainly reflects the glycemic status of patients with T2DM during the past 2–3 months (32). FPG has a moderate sensitivity to hyperglycemia (33) and 2h-PPG is an independent predictor of diabetes mellitus (34). Both are used to assess the current glycemia levels, the former being associated with basal insulin secretion from pancreatic β-cells and glucose production in the liver (34), and the latter reflecting postprandial insulin secretion (35). Meta-analysis showed that dorzagliatin significantly reduced HbA1c, FPG, and 2h-PPG compared with placebo, and TSA confirmed that these benefits were conclusive. This implies that dorzagliatin has a clear hypoglycemic effect, both in terms of short-term glycemic control and long-term glycemic modulation. Meta-analysis results also showed that dorzagliatin had an increase in HOMA2-β and a decrease in HOMA2-IR, and TSA confirmed the benefit of HOMA2-β. HOMA2-β and HOMA2-IR can assess alterations in pancreatic β-cells function and insulin resistance, respectively (36, 37). This evidence suggests that dorzagliatin modulates pancreatic β-cells function and reduces insulin resistance, a result consistent with the findings of Chow et al. (38) and Feng et al. (39), and islet β-cells dysfunction and insulin resistance are central links of T2DM (27). In addition, Zhu et al. (18) and Yang et al. (19) found that dorzagliatin still had significant reductions in HbA1c, FPG, and 2h-PPG at 52 weeks of continuous administration, suggesting that continued administration of dorzagliatin can achieve at least 52 weeks of hypoglycemic benefit without complete loss of efficacy over time, as was the case with AZD-1656. Zhu et al. (17) found that HOMA2-IR scores remained significantly lower at the 13th week after dorzagliatin was discontinued, and Zeng et al. (40) also found that patients with T2DM maintained stable glycemic control after discontinuation of dorzagliatin (7). This suggests that the effects of dorzagliatin in lowering glycemia and improving insulin resistance have long term effects, which implies that dorzagliatin may have a higher clinical value. Notably, the findings of Yang et al. (19) were based on the context of combined metformin rather than dorzagliatin alone. Fortunately, the study by Chen et al. (41) confirmed that there is no interaction between dorzagliatin and metformin, so there is no need to be concerned about the effect of metformin on the efficacy of dorzagliatin.

On blood pressure-related endpoints, meta-analysis showed no significant differences in SBP and DBP for dorzagliatin compared with placebo, suggesting that dorzagliatin does not have a risk of elevated blood pressure. On blood lipid-related endpoints, meta-analysis showed that dorzagliatin increased TG by 0.43 mmol/L and TC by 0.13 mmol/L compared with placebo, and TSA confirmed that the differences in TG and TC were conclusive. Notably, although dorzagliatin caused a mild increase in TG and TC, the increase was significantly lower than previous GKA (42) and did not increase the risk of hyperlipidemia. In addition, dorzagliatin does not cause an increase in LDL-C and a decrease in HDL-C, with LDL-C being precisely one of the main causes of increased major cardiovascular adverse events (43, 44). Overall, dorzagliatin has the advantage of not causing an increase in LDL-C and a decrease in HDL-C, and the disadvantage of having a potential risk of increasing TG and TC. Clinicians should remain aware of the potential risk of increased TG with dorzagliatin, as elevated TG is a typical manifestation of T2DM and increases the cardiovascular risk of patients. We look forward to more studies exploring the cardiovascular risk of dorzagliatin in the future.

On weight-related endpoints, dorzagliatin increased body weight by 0.38 kg and BMI by 0.14 kg/m^2^ compared with placebo. TSA confirmed that these differences are conclusive, which seems to imply that dorzagliatin has an increased risk of body weight. Interestingly, both included studies reported a trend in reduction of body weight and BMI in dorzagliatin's own pre and post controls, although this reduction was not statistically different. The study by Yang et al. (19) showed that dorzagliatin treatment reduced patients' body weight by 0.19 kg and BMI by 0.07 kg/m^2^, and the study by Zhu et al. (18) study found that dorzagliatin treatment reduced patients' weight by 0.14 kg and BMI by 0.05 kg/m^2^. There are two possible reasons for this situation. Firstly, the effect of dorzagliatin on body weight gain was so weak, and the psychological effect of placebo exceeded that of dorzagliatin. Secondly, throughout the treatment period, the researchers could not fully control the patients' diet as well as their lifestyle habits, etc. These confounding factors may have contributed to the differences in the results. Therefore, it is not certain that dorzagliatin has a risk of increasing body weight and BMI. In addition, although both meta-analysis and TSA judged the body weight gain of dorzagliatin relative to placebo to be statistically different, it was not clinically significant enough, as the body weight gain of 0.38 kg over 24 weeks was extremely minimal. Therefore, we can tentatively conclude that the weak effect of dorzagliatin on body weight does not pose a significant potential risk.

On safety endpoints, total AEs were significantly higher for dorzagliatin than for placebo, while serious AEs, hyperlipidemia, and hypoglycaemia were comparable to placebo, and TSA showed none of these results to be conclusive. Although the study results showed significantly higher total AEs for dorzagliatin than for placebo, none of the drug-related AEs in the included studies was reported separately at a higher rate than that of placebo, and the difference in total AEs may be the result of the accumulation of multiple indicators. In previous drug studies, hypoglycemia as well as elevated triglyceride levels were the major AEs in GKA. Whereas, this study confirmed that dorzagliatin triggered only 0.7 and 0.4% of drug-related hypoglycemia and hyperlipidemia, suggesting that dorzagliatin does not significantly increase the incidence of hypoglycemia and hyperlipidemia. In addition, meta-analysis confirmed that dorzagliatin does not increase serious AEs and related studies showed that dorzagliatin can be used in patients with T2DM at all stages of renal impairment (12, 45), which implies that dorzagliatin has a good safety profile.

Although this study strictly followed the PRISMA guidelines for systematic reviews and meta-analysis methods, the study still had some limitations (as follows). (1) A total of 3 clinical trials and a sample size of 1,332 were included in this study, with a small study base and total sample size, which may lead to reduced precision. (2) Narrow inclusion criteria may limit the generalizability of the results. All studies excluded patients with abnormal liver and kidney function, which means that the results of meta-analysis are not applicable to patients with hepatic impairment or renal insufficiency. Moreover, Zhu et al. (18) subjectively excluded patients with a history of T2DM for more than 3 years, and Yang et al. (19) excluded patients with TG >5.7 mmol/L, and these factors can lead to a decrease in generalizability of the results. (3) All of the three included studies were conducted in China and the study population was predominantly of Chinese descent. This implies that the benefit of dorzagliatin may only apply to Chinese or Asians, and it is unclear how dorzagliatin works in European-Americans and Africans. (4) The efficacy endpoints of the included studies were all at 24 weeks, meaning that the results of the studies only reflect the short-term or medium-term efficacy of dorzagliatin. Of concern is that while dorzagliatin is beneficial in the early stages after meal ingestion and absorption, it is more important to further utilize glucose for energy production and fatty acid synthesis and to incorporate it into fat acid storage tissues. Overweight or obesity, a prominent feature of T2DM, may limit the insulin secretory mechanisms of glycemic control, meaning that the long-term efficacy of dorzagliatin is unclear and requires continued exploration by more new studies.

In view of the limitations of the existing studies, we expect that future studies will be continuously improved. Firstly, continue to conduct similar randomized controlled double-blind trials. This could increase the samples and clinical data available for inclusion in evidence-based medicine, which could further improve the precision of study results. Secondly, a stratified study can be conducted controlling for relevant variables. The effect of dorzagliatin on patients with T2DM of different ages, disease duration, gender, and basal weight can be explored in a stratified manner in order to assess the full range of drug action characteristics on different baseline populations. Thirdly, to establish research centers in European-American and African countries. It is possible to further understand the effects of dorzagliatin in European-Americans and Africans, as well as to comprehensively assess the benefits and potential risks of dorzagliatin use in different races in combination with published studies. Fourthly, the value of dorzagliatin in combination medication can be studied in depth. As T2DM progresses, most patients need to achieve stable glycemic control with combination medications, and even eventually need to be treated with insulin (46). Fifthly, although theoretically the effect of dorzagliatin in raising TG and TC may pose a potential cardiovascular risk, there are no studies exploring major cardiovascular adverse events with dorzagliatin. We look forward to future studies that continue to explore the potential effects of dorzagliatin on cardiovascular events. Therefore, it is essential to actively conduct clinical trials of dorzagliatin in combination with other hypoglycemic agents or insulin, and a comprehensive assessment of the benefits and risks of dorzagliatin in combination is a prerequisite for rational clinical use. We expect dorzagliatin-related clinical trials to be further optimized and look forward to the benefits of dorzagliatin for patients with T2DM.

## Conclusion

In terms of efficacy, dorzagliatin is effective in lowering glycemia, reducing insulin resistance and improving pancreatic β-cells function, with good glycemic control, without affecting blood pressure, LDL-C, and HDL-C. Although dorzagliatin caused a mild increase in TG and TC, it did not increase the incidence of hyperlipidemia, and the small increases in body weight and BMI were not clinically significant enough. In terms of safety, the total AEs caused by dorzagliatin may be a cumulative effect of single AEs, with no drug-related adverse event being reported at a higher incidence than that of placebo alone. Dorzagliatin's serious AEs, hyperlipidemia, and hypoglycemia are comparable to those of placebo, with a good safety profile. Current clinical trials have been conducted with dorzagliatin alone or in combination with metformin, and the role of dorzagliatin in other combinations needs to be further explored.

## Data availability statement

The original contributions presented in the study are included in the article/supplementary material, further inquiries can be directed to the corresponding author.

## Author contributions

YY conceived and designed the study. KT and XY participated in data processing and statistical analysis. YY, GH, and SY drafted the manuscript. FZ, PJ, MZ, and WJ contributed to data analysis and interpretation. YY, XY, KT, and WJ supervised the review of the study. All authors seriously revised and approved the final manuscript.

## Funding

This study was funded by the National Natural Science Foundation of China (81973753).

## Conflict of interest

The authors declare that the research was conducted in the absence of any commercial or financial relationships that could be construed as a potential conflict of interest.

## Publisher's note

All claims expressed in this article are solely those of the authors and do not necessarily represent those of their affiliated organizations, or those of the publisher, the editors and the reviewers. Any product that may be evaluated in this article, or claim that may be made by its manufacturer, is not guaranteed or endorsed by the publisher.
